# Supplementary material for: Dichloroacetate and Quercetin Prevent Cell Proliferation, Induce Cell Death and Slow Tumor Growth in a Mouse Model of HPV-Positive Head and Neck Cancer
Source: Cancers (Basel). 2024 Apr 17;16(8):1525. doi: 10.3390/cancers16081525 (PMC11048222; doi:10.3390/cancers16081525)
Supplement: Supplementary file 1 [file cancers-16-01525-s001.zip › cancers-2776568-supplementary.pdf]

Supplementary Figures:

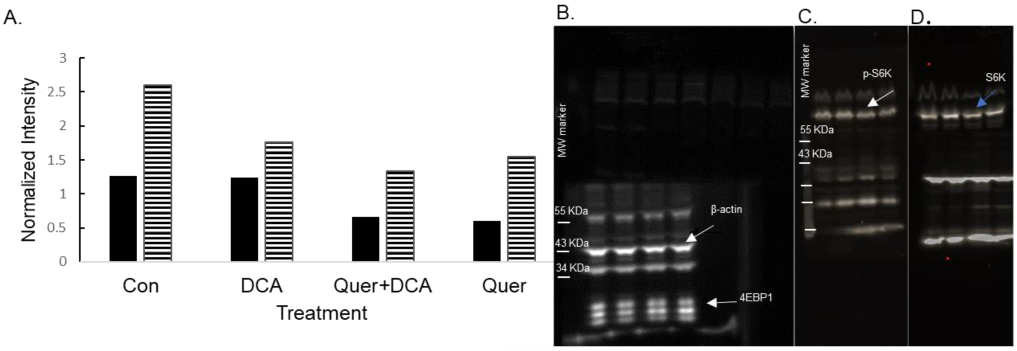

Figure. S1. A) Quantification using Image J of the mean band intensity from Figure 1. Mean band intensity of pS6 and 4EBP1 relative to the mean band intensity of the house keeping gene β-actin. B) Image of original membrane probed for 4EBP1 and β-actin. C) Image of membrane probed with anti-pS6K. D) Membrane probed with anti-S6K.

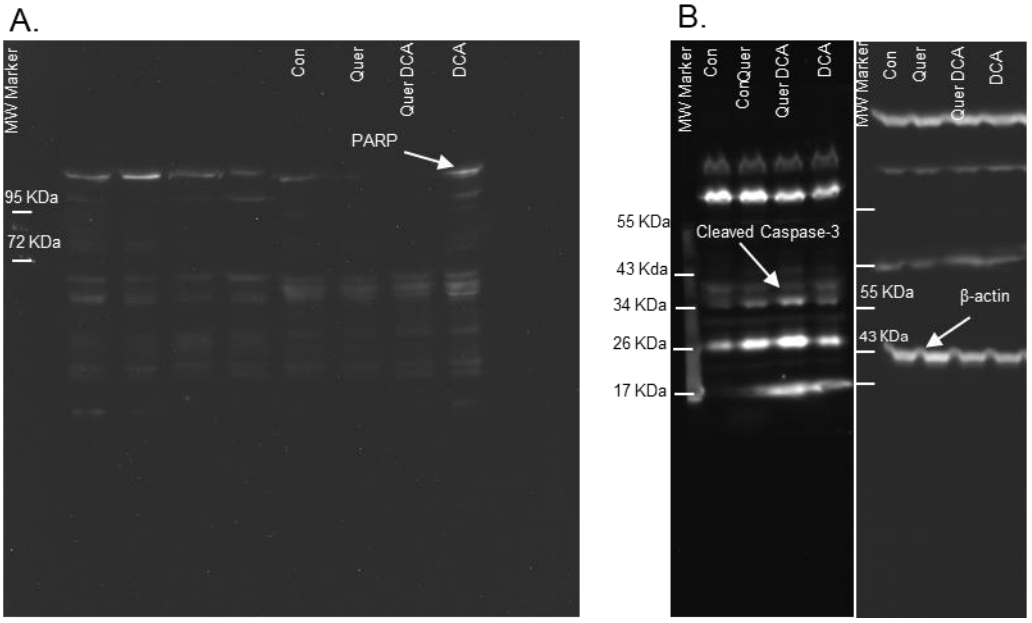

Figure S2. Original western blots of western blot depicted in Fig.2. A) Arrow indicates presence of PARP following drug treatments specified in Fig. 2. B. Expression of caspase-3 and β-actin (indicated by arrowhead) following treatment of drugs.

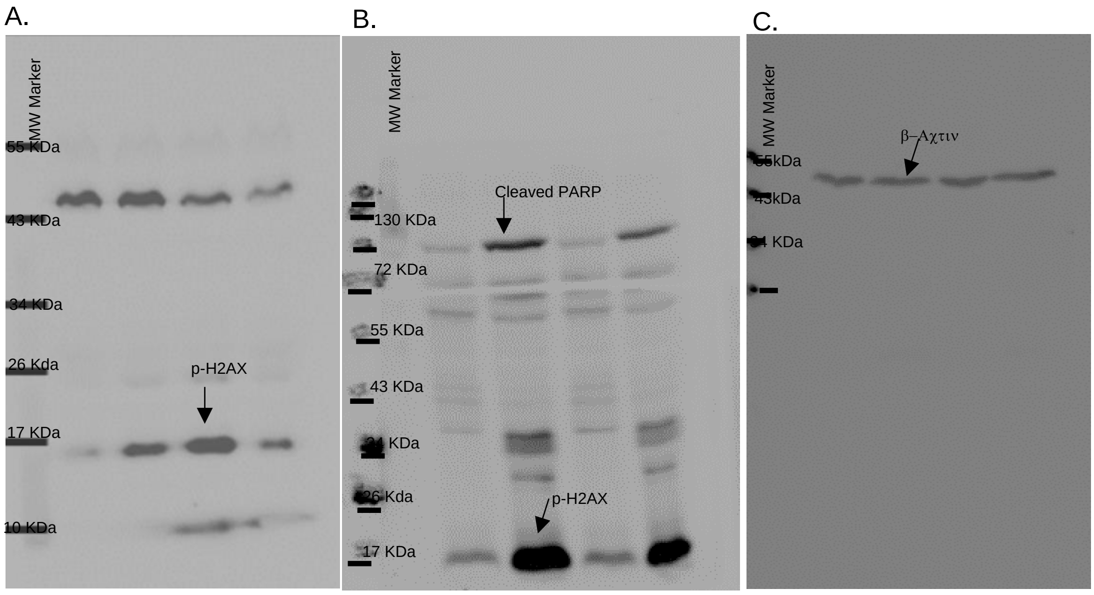

Figure S3. Original membrane for western blots illustrated for Fig 3c. A) p-H2AX band indicated with arrowhead. B) Cleaved PARP and pH2AX indicated by arrowhead. C) Housing keeping gene β-Actin indicated by arrowhead.
